# Supplementary material for: Genomic Divergence Characterization and Quantitative Proteomics Exploration of Type 4 Porcine Astrovirus
Source: Viruses. 2022 Jun 24;14(7):1383. doi: 10.3390/v14071383 (PMC9319226; doi:10.3390/v14071383)
Supplement: Supplementary file 1 [file viruses-14-01383-s001.zip › Supplementary Data S1.pdf]

Table S1. The PCR primers for detection of porcine astrovirus.

Name	Primer Sequences (5′-3′)	Length (bp)
PAstV-F	GGATTTACAGTTGGCCCAGAT	249
PAstV-R	CCTGTCCATCTGCCTTTCTGT	

Table S2. The primers for PCR amplification of the complete genome of PAstV/SH/2022/CM1 strain.

Name	Primer Sequences	Location (nt)	Length (bp)
T1-R	TAAGTGMTGYTGCAATTGG	985–1003	
T2-F	TTATCTDGCAGCRGCMTCYTT	867–887	1480
T2-R	CGTTYTGRTAGTAAGATGGTAT	2326–2347	
T3-F	GAAGGGTAAGACCAAGCATGGC	2045–2066	1034
T3-R	ATTGCTGAAAAGGCAGACACATAGG	3055–3079	
T4-F	TTGATTCAACACCRGCTTAT	2974–2993	1950
T4-R	CACCAGGTTTKGAATRDATTTT	4903–4924	
T5-F	GGACCWAAGGAKGGYTGGTWT	4666–4686	1666
T5-R	GCATAATCATCATCATCCAART	6311–6332	
T6-F	CCAGGAATTRGGTTGARTTTG	6221–6241	
